# Age-Dependent Decline in Skeletal Muscle Oxygenation Revealed During Intermittent Hypoxia and Recovery

**DOI:** 10.1093/geroni/igaf122.3531

**Published:** 2025-12-31

**Authors:** Stefano Donega, Kenneth W Fishbein, Rolando J Hernandez, Ross McDevitt, Cynthia Chisolm, Martina Rossi, Mirko Baranzini, Luigi Ferrucci

**Affiliations:** NIA/NIH Biomedical Research Center, Baltimore, Maryland, United States; National Institute on Aging, Baltimore, Maryland, United States; National Institute on Aging, Baltimore, Maryland, United States; National Institute on Aging, Baltimore, Maryland, United States; NIA, Baltimore, Maryland, United States; NIA/NIH, Baltimore, Maryland, United States; National Institute on Aging, Baltimore, Maryland, United States; NIA, Bethesda, Maryland, United States

## Abstract

Aging and age-associated syndromes such as peripheral artery disease have been previously associated to impaired mitochondrial aerobic metabolism and to ultimately contribute to sarcopenia. The mechanisms underlying this decline are still unclear, partly because existing indirect methods - such as near-infrared spectroscopy - lack the required resolution to accurately assess intramuscular dynamics. In this study, we measured hamstring muscle oxygenation in 34 C57BL/6 mice, clustered as young (∼4 months), adult (∼11 months), and old (∼21 months). Intramuscular oxygen concentration was measured with a fiber-optic probe at a rate of 1 sample per second, alongside continuous monitoring of vital signs under isoflurane anesthesia at three stages during: (I) 1:30 minutes baseline, (II) five 2:30 minutes cycles of acute intermittent hypoxia (21–5% O_2_), and (III) 1:30 minutes recovery at normoxia (21% oxygen). Baseline oxygenation declined significantly with age and the difference persisted across all five IH cycles. Age-impairments were mostly pronounced during the reoxygenation phase, where older mice showed moderated curve slopes and lower zenith values across all cycles without differences in the kinetics of oxygen depletion during the descending phase, indicating age-preserved hypoxic response. Oxygen levels during recovery remained significantly reduced with age. Our findings reveal a robust age-dependent decline in skeletal muscle oxygenation not only during a IH stress, but also at baseline and recovery, offering new tool and insights to investigate impaired oxygen dynamics in aging and muscle dysfunction.

